# Exploring Artificial Intelligence-Generated Feedback for Pharmacy Students †

**DOI:** 10.3390/pharmacy14070147

**Published:** 2026-10-01

**Authors:** Diyaben Patel, Mariam Abouelsaad, Jason B. Reed, Amy Heck Sheehan

**Affiliations:** 1 Department of Pharmacy, Union Hospital, Terre Haute, IN 47804, USA; diyaptl276@gmail.com; 2 Department of Pharmacy Practice, College of Pharmacy, Purdue University, West Lafayette, IN 47907, USA; mariamabouelsaad1@gmail.com (M.A.); reed252@purdue.edu (J.B.R.); 3 Libraries and School of Information Studies, Purdue University, West Lafayette, IN 47907, USA

**Keywords:** AI-assisted feedback, artificial intelligence, journal club, literature evaluation, peer-review, pharmacy education

## Abstract

Background: The integration of artificial intelligence (AI) into educational environments is accelerating, with growing interest in its potential to enhance learning outcomes. This pilot study aimed to assess student perceptions regarding the quality and educational impact of AI-generated feedback. Methods: Second-professional-year pharmacy students (n = 148) enrolled in a drug literature evaluation course received AI-generated feedback for a draft assignment which required students to critically evaluate a recently published journal article. Feedback was generated by an AI assistant that offered iterative feedback based on the assignment rubric. After the activity, students completed an electronic survey instrument evaluating the AI feedback regarding its alignment with rubric criteria, accuracy, clarity, and tone. Student perceptions of the feedback’s impact on learning were also assessed. Results: A total of 148 students (100%) completed the activity and responded to at least one item of the survey tool. Most students agreed with the AI-generated feedback for each section of the assignment in regard to clarity (61.5% to 76.2%) and tone (66.9% to 74.3%), with lower agreement related to alignment with the rubric (33.8% to 60.4%) and accuracy (34.5% to 54.2%). Students also expressed concern about the reliability of AI feedback (71.2%) and risk for plagiarism (50%). Conclusions: AI-generated feedback was positively received in some areas. However, students expressed apprehension regarding its accuracy, reliability, and potential accusation of plagiarism. These findings suggest further refinement is necessary to enhance AI trustworthiness and educational value in academic settings.

## 1. Introduction

Artificial intelligence (AI), including large language models (LLMs) such as ChatGPT, is beginning to influence pharmacy education and practice in visible ways [1,2,3]. A recent survey reported that 58% of pharmacy students reported use of AI for academic work [4]. This is a substantial increase from only 20 to 30% of pharmacy students reporting use of AI for academic purposes in 2023 [5,6]. While the literature has reported that pharmacy students are somewhat apprehensive about incorporating AI into learning activities due to concerns about accuracy, bias, ethical use, and loss of critical thinking skills, most students express a positive outlook on the potential applications of AI and are interested in learning how to effectively use AI to support their success [4,6,7,8,9,10]. Pharmacy students report using AI tools to summarize readings, retrieve drug information, clarify difficult concepts, and generate study materials [6]. One area where AI may be particularly useful is in providing automative feedback for written work. Prior research in higher education suggests that artificial intelligence (AI)-generated feedback can provide rapid, scalable, formative feedback for rubric-based written assignments [11,12]. However, it has also been noted that learner perceptions of trustworthiness and accuracy of automated feedback strongly influence whether they choose to engage with or apply the feedback [13,14]. Students learn within increasingly complex digital environments characterized by continuous exposure to online information, social media content, and rapidly evolving artificial intelligence technologies. As a result, issues surrounding information quality, trust, and digital literacy may influence how learners perceive and utilize AI-generated educational support.

Feedback is a significant factor related to positive student learning outcomes [15]. Formative feedback on written work is a primary mechanism for developing students’ skills in drug information, critical appraisal, and scholarly communication, particularly in journal club assignments. However, providing students with detailed and constructive feedback on written work in a timely manner can be a major challenge for most faculty, another factor that may make AI-generated feedback an attractive solution.

Limited research has examined how pharmacy students perceive AI-generated feedback on their own writing, how much they trust it, or how they integrate it into their revision process [14]. Most existing studies focus largely on AI use patterns, attitudes, and content accuracy rather than on students’ experiences receiving and acting on AI-generated formative feedback [4,5,6,7,8,9,10,16]. This gap is especially important as programs explore AI-assisted feedback for tasks such as literature evaluation and journal club critiques, where the quality of feedback directly shapes students’ reasoning and professional identity. Given limited research, and the fact that student perceptions play an important role in their decision to engage with AI-generated feedback, the present pilot study explores the use of AI-generated feedback in a drug literature evaluation. This Brief Report first describes the implementation of an AI-generated feedback activity, followed by an assessment of student perceptions regarding the quality and educational value of the feedback, and concludes with implications for future integration of AI-supported educational technologies.

## 2. Materials and Methods

### 2.1. Charlie AI Activity

The instructional technology team at our institution developed an AI writing assistant, known as Charlie AI, designed to provide rubric-based feedback on student assignments. The AI assistant is powered by OpenAI’s GPT-4 model and offers iterative feedback based on the specific assignment rubric, allowing students to refine and resubmit their work while maintaining data privacy. A pilot active learning exercise using Charlie AI was incorporated into a written assignment for a second-professional-year, required drug information and literature evaluation course. The assignment required critical evaluation of a recently published journal article with respect to ten different sections: journal and authors, study subjects, study design, endpoints, statistical analysis, results, overall strengths, overall limitations, generalizability of results, and clinical impact (rubric provided in the Supplementary Materials). The assignment had a two-page limit. Students submitted their draft written assignment one week prior to the due date and Charlie AI provided feedback on each section of the assignment rubric. Students reviewed the AI-generated feedback during a designated class period and were permitted to discuss their feedback with their peers and the course instructors. After the activity students had the opportunity to revise their draft based on Charlie AI’s feedback before submission of the final assignment. The overall objective of this in-class activity was to foster critical thinking skills and help students understand the strengths and limitations of AI. This course includes three journal club assignments distributed through the semester. Historically, students enrolled in this course participate in three in-class peer-review sessions for the journal club assignments, during which time they receive electronic, anonymous feedback on their draft assignment from three of their classmates and provide feedback to three other classmates. Our experience with this peer-review activity has demonstrated enhanced learning behaviors, increased interest in peer-review, and improved self-competence regarding literature evaluation [17,18]. In this pilot, the traditional peer-review activity associated with the third and final journal club assignment was replaced with the Charlie AI feedback activity.

### 2.2. Assessment of the Activity

Immediately following the in-class Charlie AI activity, students were asked to complete an electronic survey instrument regarding their perceptions of the quality of Charlie AI-generated feedback and perceptions of the overall impact of Charlie AI on learning. The survey instrument consisted of two sections and is provided for review in the Supplementary Materials. The first section of the survey instrument asked students for their level of agreement (yes, partially, or no) with the Charlie AI feedback in relation to its alignment with the assignment rubric, accuracy, clarity, and tone for each of the assignment sections. The second section of the survey instrument collected student perceptions of using Charlie AI-generated feedback and included selected questions from the validated TAME-ChatGPT survey tool for assessing health-care students’ attitudes towards ChatGPT [19]. Students were provided with approximately 15 minutes at the end of the class period to complete the survey tool and were awarded 5 points for participation. Survey responses were anonymous and confidential. The study was determined to be exempt research by the institution’s Investigational Review Board (IRB2024-1674). Data are presented descriptively.

## 3. Results

One-hundred and forty-eight second-year pharmacy students reviewed the Charlie AI-generated feedback for their written draft assignment during class, with the opportunity to edit their draft before submitting their final assignment. All students enrolled in the course (100%, 148/148) completed at least one of the survey instrument items. Table 1 shows the results of student agreement with Charlie AI’s feedback in relation to its alignment with the assignment rubric, accuracy, clarity, and tone for each section of the assignment. Levels of agreement with Charlie AI-generated feedback varied across journal club rubric domains, with most students agreeing with the AI-generated feedback for each specific section of the assignment in regard to clarity (61.5% to 76.2%) and tone (66.9% to 74.3%), with lower agreement related to alignment with the rubric (33.8% to 60.4%) and accuracy (34.5% to 54.2%). Student agreement was lowest for the accuracy of feedback related to the more nuanced aspects of study evaluation such as the overall limitations (39.9%, 57/143) and generalizability of study results (34.5%, 49/142).

Table 2 presents student perceptions regarding use of Charlie AI’s feedback and impact on learning. Concerns regarding the reliability and risks associated with AI-generated feedback were common among respondents. Approximately half of respondents indicated concern about potential plagiarism accusations associated with use of the AI tool. Participants were split with regard to perceived learning benefits from the AI-generated feedback, with 50.7% (74/146) of students indicating that they learned something new from the feedback, and 46.6% (68/146) reporting that the feedback supported their critical thinking. Only one-third of students agreed that the Charlie AI feedback improved their final assignment grade. Student intentions to continue using the Charlie AI feedback tool were also mixed but trended toward reluctance. More than half of respondents reported that they would not choose to use the Charlie AI tool again in future journal club assignments. Similarly, 50.7% (74/146) of students did not support integrating the Charlie AI feedback tool into future courses. Finally, when asked to identify preferred sources of feedback, students most frequently selected their peers (57.5%, 84/146) or a teaching assistant/instructor (39.7%, 58/146). Only 2.7% of students (4/146) indicated a preference for Charlie AI-generated feedback.

## 4. Discussion

Use of AI-generated feedback for journal club assignments in a drug literature evaluation course received mixed reviews from second-year pharmacy students. While a small majority of students agreed with the specific feedback in relation to alignment with the rubric, clarity, and tone, they expressed apprehension regarding its accuracy, reliability and potential risk for accusation of plagiarism. Students indicated preference for peer feedback over the AI-generated feedback and were hesitant to support further integration of Charlie AI tools into their coursework.

Previous perception studies suggest that pharmacy and other health profession students generally view AI as promising for learning, while simultaneously expressing concerns about accuracy, bias, and ethical use [4,6,7,8,9,10]. In a recent pilot study, Pellesi evaluated pharmacy students’ trust in AI-generated feedback through the use of Microsoft Copilot to provide feedback for a master’s-level evidence-based medicine course for pharmacy students in Denmark [14]. Student groups participated in peer-feedback activities with the additional option of receiving AI feedback. Groups that chose not to use AI feedback indicated lack of trust and concerns about the quality of AI-generated feedback as reasoning for not using AI. Among student groups who opted in to receive AI feedback, the majority rated peer feedback as more valuable than AI feedback. Reasons for this preference included lack of depth, generalized comments that failed to acknowledge discipline-specific nuances, and redundant comments from AI-assisted feedback. Our findings are consistent with these results in that the majority (71%) of our students were concerned about the reliability of AI feedback and 90% preferred to receive feedback from a human (peer, teaching assistant, or instructor) over AI-generated feedback. Notably, only 30% of students agreed that using AI feedback would improve their grade or that the feedback helped their confidence in critiquing journal articles. In contrast, the previous literature evaluating the impact of peer-feedback activities in pharmacy students has reported positive perceptions including improved self-competence [18,20]. This also aligns with the majority of students in this pilot preferring peer feedback over AI-generated feedback. More research and refinement of AI-generated feedback is needed before AI can replace human feedback.

One of the possible reasons why Charlie AI was not viewed positively by the students in this pilot study could be because it was originally designed and trained for longer form written assignments, such as essay papers in the social sciences. One of the key components to the journal club assignment is the two-page limit. Charlie AI was designed to always provide iterative suggestions for improvement. This resulted in some students sharing a frustration that even if a particular section of the assignment was complete and accurate, Charlie AI would still suggest adding more detail. Charlie AI is powered by OpenAI’s GPT-4 model and generates assignment-specific feedback through rubric-guided prompting, rather than domain-specific training on prior student submissions or grading records. Perhaps further revision of the assignment rubric, improved prompt engineering, and work with the development team to train Charlie for this assignment could improve the quality of feedback. An AI tool specifically designed for literature evaluation could be perceived as more effective and useful by pharmacy students.

Student concerns regarding the reliability of AI-generated feedback may reflect broader challenges associated with evaluating information credibility in contemporary digital environments. Apprehension could also be due to the fact that students had become comfortable receiving peer feedback for the first two in-class activities. Perceptions of AI-generated feedback may have been shaped by prior exposure to peer and instructor feedback. With the peer-feedback component, each student provides feedback for three other examples of the journal critique. Regardless of the feedback received from their peers, they have the opportunity to peer-review other students’ drafts and may notice components they omitted from their own draft. The act of reviewing the work of their peers may expose them to new ideas to incorporate into their own assignment and encourage them to think more critically about the critique and the rubric. This component is missing from AI feedback. The present study did not evaluate the relative contributions of reviewing peers’ work versus receiving AI-generated feedback. Student perceptions may differ in a course environment where AI-generated feedback served as the primary or sole source of formative feedback. Future studies should compare AI-only, peer-review, and hybrid feedback models to better understand their relative educational value.

The results of this pilot are limited to a single class and a single activity at one College of Pharmacy. The Charlie AI tool was originally designed for use in classes with longer written assignments, such as essay papers, which may have affected the feedback provided. It should be noted that this pilot did not independently assess the objective accuracy of Charlie AI-generated feedback. Therefore, survey responses related to accuracy reflect student perceptions rather than verified measures of correctness. The survey instrument also did not collect qualitative data regarding the reasons underlying students’ responses. Future studies incorporating interviews, focus groups, or open-ended survey questions may provide greater insight into factors influencing student trust, acceptance, and use of AI-generated feedback. In addition, many confounding variables could have affected the student perceptions of quality of the feedback including the complexity of the specific clinical trial chosen for the activity, students’ confidence in their own work, their ability to independently assess the validity of the feedback received, the prompts used based on the rubric, and the completeness of the student’s draft assignment, with more complete or high-quality drafts perhaps receiving less useful feedback compared to other drafts. Finally, measures related to critical thinking, reliability, and grade improvement reflect student perceptions and beliefs about AI-generated feedback rather than objective measures of educational outcomes. Future studies should evaluate these outcomes using direct measures of learning, assignment performance, and critical thinking skills.

## 5. Conclusions

While Charlie AI provided rubric-based feedback and was positively received in some areas, students expressed apprehension regarding its reliability, the potential accusation of plagiarism, and lack of improvement in their grade or self-confidence. Most students preferred peer or instructor feedback and were hesitant to support further integration of Charlie AI into their coursework. These findings suggest that while AI has potential as a supplemental feedback tool, further refinement is necessary to enhance its trustworthiness and educational value. Because these findings reflect student perceptions rather than objective measures of learning outcomes or feedback quality, additional research is needed to evaluate the educational effectiveness of AI-generated feedback and to better understand the factors influencing student trust and acceptance of AI-assisted learning.

This article is a revised and expanded version of “Assessing Student Perceptions of Artificial Intelligence Generated Feedback”, which was presented as a trainee poster at the American Association of Colleges of Pharmacy 2025 Pharmacy Education meeting in Chicago, IL, on 21 July 2025.

## Figures and Tables

**Table 1:** Student agreement with Charlie AI feedback.

Assignment Component	Perceived Alignment with Rubric Does the Feedback Align with the Rubric’s Requirements?			Perceived Accuracy Is the Feedback Factually Correct?			Perceived Clarity Is the Feedback Clear and Understandable?			Perceived Tone Does Charlie AI Always Use Positive, Growth-Mindset Language to Encourage Students?		
	Yes n (%)	Partially n (%)	No n (%)	Yes n (%)	Partially n (%)	No n (%)	Yes n (%)	Partially n (%)	No n (%)	Yes n (%)	Partially n (%)	No n (%)
**Journal &** **Authors ^1^**	66/149 (44.3%)	74/149 (49.7%)	9/149 (6.0%)	72/147 (48.9%)	65/147 (44.2%)	12/147 (8.2%)	109/149 (73.2%)	30/149 (20.1%)	10/149 (6.7%)	107/149 (71.8%)	30/149 (20.1%)	12/149 (8.0%)
**Study Subjects**	77/147 (52.4%)	60/147 (40.8%)	10/147 (6.8%)	8/147 (46.3%)	59/147 (40.1%)	20/147 (13.6%)	104/147 (70.7%)	31/147 (21.1%)	12/147 (8.2%)	101/147 (68.7%)	34/147 (23.1%)	12/147 (8.2%)
**Study Design**	84/143 (58.7%)	51/143 (35.6%)	8/143 (5.6%)	71/142 (50.0%)	54/142 (38.0%)	17/142 (12.0%)	109/143 (76.2%)	28/143 (19.6%)	6/143 (4.2%)	105/142 (73.9%)	30/142 (21.1%)	7/142 (4.9%)
**Endpoints**	84/143 (58.7%)	42/143 (29.4%)	17/143 (11.9%)	69/143 (48.3%)	61/143 (42.7%)	13/143 (9.1%)	106/143 (74.1%)	30/143 (21.0%)	7/143 (4.9%)	105/143 (73.4%)	29/143 (20.3%)	9/143 (6.3%)
**Statistical** **Analysis**	88/144 (61.1%)	43/144 (29.9%)	13/144 (9.0%)	78/144 (54.2%)	46/144 (31.9%)	20/144 (13.9%)	102/144 (70.8%)	29/144 (20.1%)	13/144 (9.0%)	107/144 (74.3%)	27/144 (18.8%)	10/144 (6.9%)
**Results**	87/144 (60.4%)	45/144 (31.3%)	12/144 (8.3%)	78/144 (54.2%)	55/144 (38.2%)	11/144 (7.6%)	106/144 (73.6%)	29/144 (20.1%)	9/144 (6.3%)	106/144 (73.6%)	28/144 (19.4%)	10/144 (6.9%)
**Overall** **Strengths**	71/143 (49.6%)	48/143 (33.6%)	24/143 (16.7%)	59/143 (41.3%)	44/143 (30.8%)	40/143 (28.0%)	88/143 (61.5%)	33/143 (23.1%)	22/143 (15.4%)	98/143 (68.5%)	28/143 (19.6%)	17/143 (11.9%)
**Overall** **Limitations**	65/143 (45.5%)	56/143 (39.2%)	22/143 (14.4%)	57/143 (39.9%)	58/143 (40.6%)	28/143 (19.6%)	91/143 (63.6%)	32/143 (22.4%)	20/143 (14.0%)	96/143 (67.1%)	28/143 (19.6%)	19/143 (13.3%)
**Generalizability**	48/142 (33.8%)	63/142 (44.4%)	31/142 (21.8%)	49/142 (34.5%)	56/142 (39.4%)	37/142 (26.1%)	91/142 (64.1%)	34/142 (23.9%)	17/142 (12.0%)	95/142 (66.9%)	31/142 (21.8%)	16/142 (11.3%)
**Conclusion** **and Impact on Practice**	70/141 (49.6%)	52/141 (36.9%)	19/141 (13.5%)	76/141 (53.9%)	45/141 (31.9%)	20/141 (14.2%)	99/141 (70.2%)	28/141 (19.9%)	14/141 (9.9%)	98/141 (69.5%)	27/141 (19.1%)	16/141 (11.3%)

^1^ 149 responses, although 148 students enrolled in course.

**Table 2:** Student perceptions regarding use of AI feedback.

Survey Statement	Strongly Agree or Agree	Neutral	Strongly Disagree or Disagree
Concerned about the reliability of AI feedback	104/146 (71.2%)	25/146 (17.1%)	17/146 (11.6%)
Concerned about accusation of plagiarism from using feedback	73/146 (50%)	29/146 (19.8%)	44/146 (30.1%)
Concerned about privacy/security risks of using AI	36/146 (24.7%)	39/146 (26.7%)	71/146 (48.6%)
Concern using AI feedback may hurt critical thinking	45/146 (30.8%)	28/146 (19.2%)	73/146 (50%)
Appreciate the accuracy and reliability of AI feedback	49/146 (33.6%)	37/146 (25.3%)	60/146 (41.1%)
Utilizing AI feedback improves my grade	45/146 (30.8%)	38/146 (26%)	63/146 (43.2%)
Utilizing AI feedback may result in lack of originality in my critique	55/146 (37.7%)	31/146 (21.2%)	60/146 (41.1%)
Utilizing AI feedback helps critical thinking	68/146 (46.6%)	32/146 (21.9%)	46/146 (31.5%)
I plan to utilize AI feedback in future critiques	56/146 (38.4%)	38/146 (26%)	52/146 (35.6%)
Using Charlie AI would help my confidence in critiquing journal articles.	44/146 (30.1%)	35/146 (24%)	67/146 (45.9%)
I learned something new from Charlie AI’s feedback	74/146 (50.7%)	35/146 (24%)	37/146 (25.3%)
I would choose to use Charlie AI again for feedback on journal article critiques.	42/146 (28.8%)	25/146 (17.1%)	79/146 (54.1%)
I would support integrating Charlie AI as feedback tool for future classes	39/146 (26.7%)	33/146 (22.6%)	74/146 (50.7%)

## Data Availability

The raw data supporting the conclusions of this article will be made available by the authors on request.

## Supplementary Materials

The following supporting information can be downloaded at https://www.mdpi.com/article/10.3390/pharmacy14070147/s1, Figure S1. Journal Article Critique Rubric; Figure S2. Evaluating the Quality of Charlie-AI-Generated Feedback Survey Items.

## Abbreviations

The following abbreviations are used in this manuscript:

AI	Artificial Intelligence
TAME-ChatGPT	Technology-Acceptance Model Edited to Assess ChatGPT Adoption
LLM	Large Language Model
