# Supplementary material for: Exploring Artificial Intelligence-Generated Feedback for Pharmacy Students
Source: Pharmacy (Basel). 2026 Oct 1;14(7):147. doi: 10.3390/pharmacy14070147 (PMC13708426; doi:10.3390/pharmacy14070147)
Supplement: Supplementary file 1 [file pharmacy-14-00147-s001.zip › pharmacy-4471439-supplementary.pdf]

Figure S1. Journal Article Critique Rubric

0 = No information provided
1 = Incorrect information provided
2 = Omission of sufficient details
3 = Omission of minor details
4 = Complete and thorough assessment

**Journal and Authors** (evaluates journal and study authors)	0 1 2
**Study Subjects** (Evaluates whether subject characteristics are representative of patient population <u>and</u> evaluates balance of baseline characteristics between treatment groups)	0 1 2 3 4
**Study Design** (Lists study design and evaluates if study design it appropriate for study objective; evaluates appropriateness of study duration)	0 1 2 3 4
**Endpoints** (Lists the primary and secondary endpoints; evaluate the appropriateness of chosen endpoints)	0 1 2 3 4
**Statistical Analysis** (lists and evaluates appropriateness of chosen non-inferiority margin, *a priori* alpha, study power, and all statistical tests used to analyze the primary endpoint)	0 1 2 3 4
**Results** (evaluates subject consort flow diagram, list specific results for the primary endpoint; list specific safety results; assess statistical AND clinical significance of the results)	0 1 2 3 4
**Overall Strengths** (lists and describes at least 3 strengths of the study not mentioned by the authors)	0 1 2 3
**Overall Limitations** (lists and describes at least 3 limitations of the study not mentioned by the authors)	0 1 2 3
**Generalizability of Study Results** (evaluate the generalizability of study results)	0 1 2 3 4
**Final Conclusion/Impact on Practice** (state your own conclusions and comment on the impact of the study results on practice)....................................	0 1 2 3 4

Critique =_____(36 max)

SPELLING AND GRAMMAR

0 = Document contains significant (4+) grammatical and typographical errors
1 = Document contains some (1 to 3) grammatical and typographical errors
2 = Free of grammatical and typographical errors

Score =______(2 max)

REFERENCES IN PROPER FORMAT

0 = Contains significant (4+) referencing errors
1 = Contains some (1 to 3) referencing errors
2 = Free of referencing errors

Score =_____(2 max)

ARTICLE CRITIQUE IS > 2 PAGES YES NO (IF YES, SUBTRACT 4 POINTS)

TOTAL SCORE:______(40 MAX)

Figure S2. Evaluating the Quality of Charlie-AI-Generated Feedback Survey Items

Journal and Authors:

Criteria	Question	Yes	Partially	No
Alignment with journal club rubric	Does the feedback align with the rubric’s requirements?	○	○	○
Accuracy	Is the feedback factually correct?	○	○	○
Clarity	Is the feedback clear and understandable?	○	○	○
Tone	Does Charlie AI always use positive, growth-mindset language to encourage students?	○	○	○

Study Subjects:

Criteria	Question	Yes	Partially	No
Alignment with journal club rubric	Does the feedback align with the rubric’s requirements?	○	○	○
Accuracy	Is the feedback factually correct?	○	○	○
Clarity	Is the feedback clear and understandable?	○	○	○
Tone	Does Charlie AI always use positive, growth-mindset language to encourage students?	○	○	○

Study Design:

Criteria	Question	Yes	Partially	No
Alignment with journal club rubric	Does the feedback align with the rubric’s requirements?	○	○	○
Accuracy	Is the feedback factually correct?	○	○	○
Clarity	Is the feedback clear and understandable?	○	○	○
Tone	Does Charlie AI always use positive, growth-mindset language to encourage students?	○	○	○

Endpoints:

Criteria	Question	Yes	Partially	No
Alignment with journal club rubric	Does the feedback align with the rubric's requirements?	○	○	○
Accuracy	Is the feedback factually correct?	○	○	○
Clarity	Is the feedback clear and understandable?	○	○	○
Tone	Does Charlie AI always use positive, growth-mindset language to encourage students?	○	○	○

Statistical Analysis:

Criteria	Question	Yes	Partially	No
Alignment with journal club rubric	Does the feedback align with the rubric's requirements?	○	○	○
Accuracy	Is the feedback factually correct?	○	○	○
Clarity	Is the feedback clear and understandable?	○	○	○
Tone	Does Charlie AI always use positive, growth-mindset language to encourage students?	○	○	○

Results:

Criteria	Question	Yes	Partially	No
Alignment with journal club rubric	Does the feedback align with the rubric's requirements?	○	○	○
Accuracy	Is the feedback factually correct?	○	○	○
Clarity	Is the feedback clear and understandable?	○	○	○
Tone	Does Charlie AI always use positive, growth-mindset language to encourage students?	○	○	○

Overall Strengths:

Criteria	Question	Yes	Partially	No

Alignment with journal club rubric	Does the feedback align with the rubric's requirements?	○	○	○
Accuracy	Is the feedback factually correct?	○	○	○
Clarity	Is the feedback clear and understandable?	○	○	○
Tone	Does Charlie AI always use positive, growth-mindset language to encourage students?	○	○	○

Overall Limitations:

Criteria	Question	Yes	Partially	No
Alignment with journal club rubric	Does the feedback align with the rubric's requirements?	○	○	○
Accuracy	Is the feedback factually correct?	○	○	○
Clarity	Is the feedback clear and understandable?	○	○	○
Tone	Does Charlie AI always use positive, growth-mindset language to encourage students?	○	○	○

Generalizability of Study Results:

Criteria	Question	Yes	Partially	No
Alignment with journal club rubric	Does the feedback align with the rubric's requirements?	○	○	○
Accuracy	Is the feedback factually correct?	○	○	○
Clarity	Is the feedback clear and understandable?	○	○	○
Tone	Does Charlie AI always use positive, growth-mindset language to encourage students?	○	○	○

Conclusion/Impact on Practice:

Criteria	Question	Yes	Partially	No
Alignment with journal club rubric	Does the feedback align with the rubric's requirements?	○	○	○

Accuracy	Is the feedback factually correct?	○	○	○
Clarity	Is the feedback clear and understandable?	○	○	○
Tone	Does Charlie AI always use positive, growth-mindset language to encourage students?	○	○	○

The following questions are to evaluate your opinion of using Charlie AI in this course. All responses will be anonymous.

Respond to each statement using the 5-point Likert scale: 1= Strongly Disagree, 2= Disagree, 3=Neither Agree nor Disagree, 4=Somewhat Agree, 5=Strongly Agree

1. I am concerned about the reliability of the feedback provided by Charlie AI.
2. I am concerned that using Charlie AI's feedback in the future would get me accused of plagiarism.
3. I fear the potential privacy and security risks of using Charlie AI to grade assignments or provide feedback.
4. I fear using Charlie AI-generated feedback would result in me depending on AI for school assignments and not developing my critical thinking skills.
5. I appreciate the accuracy and reliability of AI-generated feedback.
6. I believe receiving Charlie AI's feedback will help me get a better grade on journal article critiques.
7. I fear relying on Charlie AI's feedback would result in a lack of originality in my journal article critiques.
8. I believe receiving Charlie AI's feedback helped me think critically about my understanding of the journal article.
9. I plan to use what I learned from Charlie AI's feedback for future journal article critiques.
10. I believe using Charlie would help me gain confidence in critiquing journal articles.
11. I learned something new from Charlie's feedback.
12. I would choose to use Charlie AI again for feedback on journal article critiques.
13. I would support integrating Charlie AI as a feedback tool for future classes.

---

14. I prefer receiving feedback from:
    a. AI/Charlie
    b. Peer review
    c. TA/Instructors
